# Physical Implausibility of Carbohydrate Ligands in Results of Deep Learning-Based Cofolding Methods

**DOI:** 10.1021/acs.jcim.5c03075

**Published:** 2026-03-23

**Authors:** Muhammad Luthfi, Adam J. Simpkin, Luc G. Elliott, Pornthep Sompornpisut, Daniel J. Rigden

**Affiliations:** † Program in Bioinformatics and Computational Biology, College of Interdisciplinary and Integrative Studies, 26683 Chulalongkorn University, Bangkok 10330, Thailand; ‡ Department of Biochemistry, Cell and Systems Biology; Institute of Systems, Molecular and Integrative Biology, 4591 University of Liverpool, Crown Street, Liverpool L69 7ZB, United Kingdom; § Center of Excellence in Computational Chemistry, Department of Chemistry, Chulalongkorn University, Bangkok 10330, Thailand

## Abstract

Stereochemistry violations in AlphaFold 3 models are more prevalent than currently appreciated. Analysis of 900 carbohydrate ligands revealed that 85.8% have errors, mainly in chirality but also including bond conversions (15.2%), planar ring distortions (3.9%), aromatic ring formations (2.5%), and improper structural configurations (0.9%). Boltz-1x reduced most violations dramatically but increased improper configurations to 22.1%, notably in *N*-acetyl-α-neuraminic acid. The BondedAtomPairs protocol reduced stereochemical issues but lost the reducing-end anomeric oxygen, highlighting ongoing challenges in accurate carbohydrate modeling.

## Graphical abstract

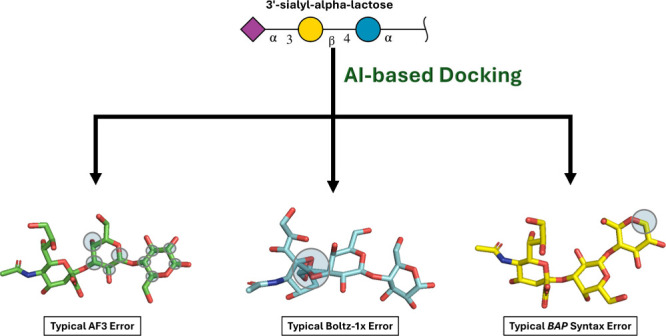

## Introduction

A significant advancement distinguishing AlphaFold 3 (AF3)^1^ from its predecessor, AlphaFold 2 (AF2),^2^ is the integration of an AI-driven cofolding feature, enabling the prediction of proteins in complex with ligands. The predictive scope of AF3 is notably extensive, encompassing not only small-molecule ligands but also other biomolecules, including proteins, nucleic acids, and glycans.^1,3^ Its overall structure performance and docking capability have been independently validated across several biomolecular classes.^4–6^ However, these benchmarks have focused primarily on protein–protein and protein–small-molecule systems. In contrast, the stereochemical fidelity of AF3 predictions — especially for carbohydrates, which possess dense chiral information and diverse ring conformations^7^— remains largely uncharacterized. This presents a critical knowledge gap, as accurate glycan modeling is essential for numerous biological processes, such as glycan-mediated signaling, immune recognition and pathogen binding, e.g., SARS-CoV-2 infection.^8^ Because of their central importance, reliable glycan modeling with AF3 could have significant implications for vaccine development and biotechnology.^9^

The original AF3 publication reported a relatively low rate of chirality violations (4.4%) but did not specify how these errors were distributed across different ligand classes.^1^ Subsequent small-scale examinations of two specific glycans, the simple linear human milk oligosaccharide lacto-*N*-neotetraose (LNnT) and the complex biantennary *N*-glycan (G2), revealed stereochemical inconsistencies upon manual inspection.^3^ However, the extremely limited sample size prevented broader conclusions. Thus, despite growing interest in AF3 cofolding capabilities, a systematic, large-scale assessment of its stereochemical accuracy for glycan modeling has not yet been performed, leaving its reliability for carbohydrate-containing complexes largely unresolved.

A separate large-scale analysis with AF3 using datasets from PLINDER and PDBbind^10,11^ employed the RDKit cheminformatics toolkit for chirality assessment. This study reported high chirality error rates for small-molecule ligands (30%–40%) but claimed that glycans, amino acids, and nucleic acids were rarely affected.^4^ However, glycans are particularly challenging since they contain far more chiral centers and linkage diversity than typical ligands, and protein-carbohydrate complexes account for only about 14 000 PDB entries (∼5.7%).^12^ The performance limitations with under-represented ligands are illustrated by recent modeling work focusing on d-peptides, a ligand class that also depends critically on correct stereochemistry, where a 51% chirality error rate in AF3 was reported.^13^ Additionally, the RDKit-based stereochemical assessment may itself introduce misclassifications.^14^ These considerations raise legitimate concerns about AF3’s ability to handle stereochemically dense molecules such as glycans. Consequently, a comprehensive and rigorous assessment of AF3’s stereochemical accuracy in glycan modeling is urgently needed to clarify its current limitations and guide future improvements. For comparison, we also test Boltz-1x, an open-source alternative to AF3, which has been reported to successfully reduce the incidence of stereochemical issues and other hallucinations by using inference-time steering.^15^ Finally, we also analyze published outputs,^3,16^ finding errors and concluding that stereochemically correct glycan modeling remains an open challenge.

## Results

### AF3-Induced Glycan Stereochemistry Violations Extend Beyond Chirality, While Boltz-1x Resolves Stereochemical Errors but Introduces New Error Types

In this work we applied AF3 and Boltz-1 to make 900 models of 15 noncovalent protein-glycan complexes (Table S1), testing different SMILES inputs (from two to four depending on availability; see Figure 3, presented later in this work) cofolding types (i.e., cognate protein-glycan, complex with nonglycan-related protein or glycan alone), and four random seeds. Manual assignment of errors of various kinds (see below) in the AF3 results revealed a significantly higher rate and diversity of stereochemistry errors than previously acknowledged. Only 14.2% of models were stereochemically correct, whereas 85.8% exhibited at least one violation. Beyond incorrect stereocenter assignment, AF3 frequently introduced additional chemically implausible features, including artificial double bonds (15.2%), planar ring distortions (3.9%), aromatic-like ring formation (2.5%), and improper monosaccharide geometries (0.9%) (Figure 1A). These violations varied in severity, ranging from a single atom to multiatom distortions within an entire monosaccharide unit. In the most severe cases, structural violations propagated across multiple monosaccharides within an oligosaccharide, indicating substantial breakdown of carbohydrate stereochemistry in AF3 predictions (Figure 1B).

**Figure 1:**
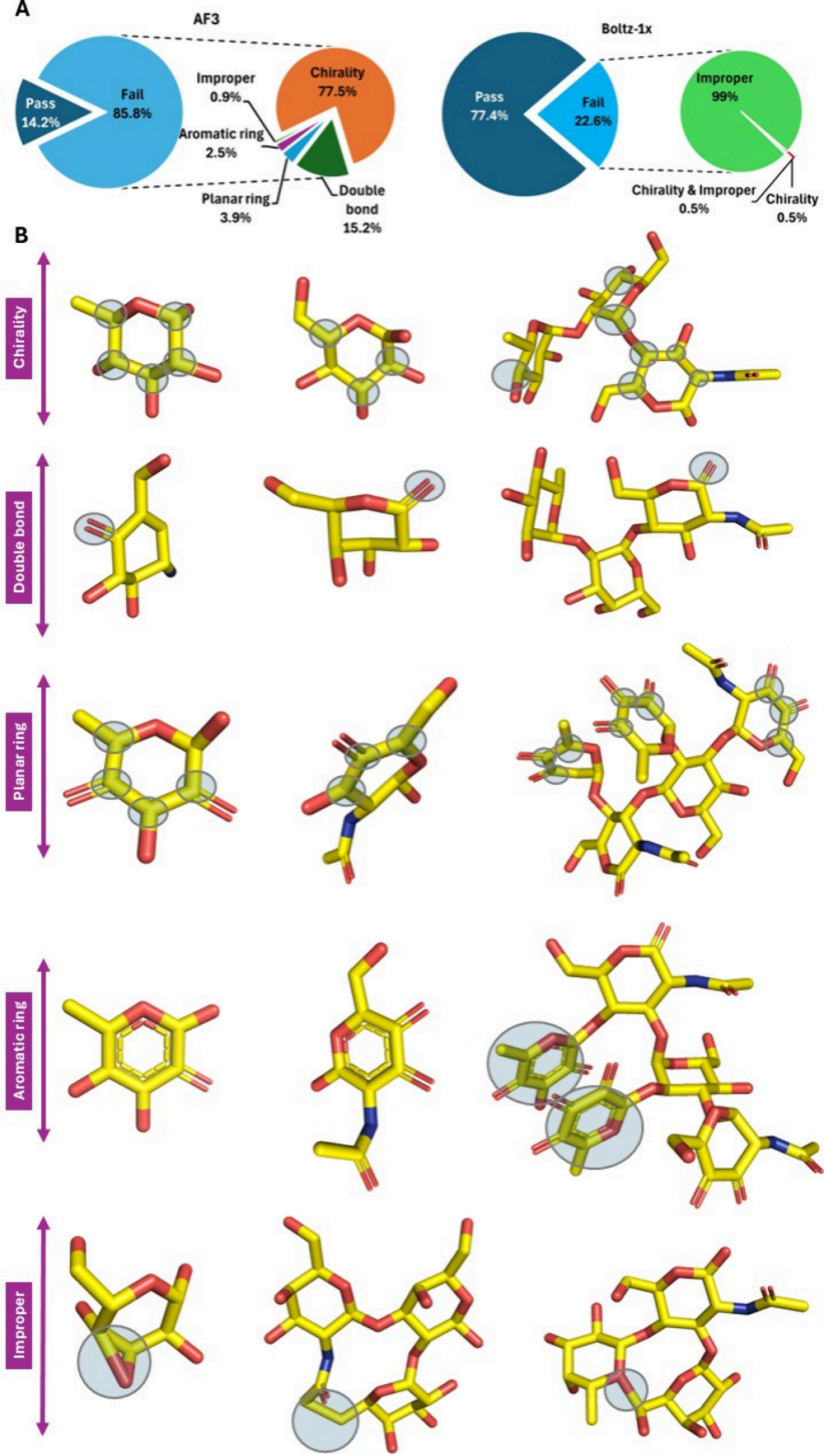
(A) Pie charts illustrating the proportion of glycan models that passed or failed stereochemical inspection, along with the distribution of error types contributing to failure. The left chart represents error proportions for AF3, while the right chart shows those for Boltz-1x. (B) Visual representation of structural violations in AF3-generated models. Blue transparent circles indicate atomic positions where deviations from the corresponding X-ray crystallographic reference structures were detected.

To our knowledge, this is the first report documenting such a wide range of stereochemical violations in glycan structures predicted by AF3. While planar ring distortions have been noted previously—for example, Ishitani and Moriwaki^4^ reported flattening of the tetrahydropyrimidine ring in ectoine—these observations were limited to isolated cases rather than systematic evaluation. In our study, we defined chirality violations as incorrect stereochemical orientation of one or more atoms or functional groups, and we classified planar ring errors when at least three atoms or functional groups within a sugar unit deviated markedly from the reference geometry. This manual assessment was backed up by Privateer analysis of a representative sample (Table S2). Even under these conservative criteria, 3.9% of AF3 models exhibited pronounced planar ring distortions, often affecting multiple sugar units within a single glycan (Figure 1B). Remarkably, nearly all units with planar ring errors also displayed aromatic-like ring features, reflecting complete loss of chirality and significant bond shortening within the ring (Figure 1B and Table 1). These results highlight severe failure mode in AF3 when handling carbohydrate ring stereochemistry.

**Table 1:** Summary of Structural Error Types Associated with Individual Monosaccharide Units Found in Ligand Oligosaccharides, Along with the Number of Ligands in the Dataset Containing Each Sugar Unit (See Also Table S1) and the Total Number of Models Analyzed^a^

Sugar Unit	Chirality	Double bond	Planar ring	Aromatic ring	Improper	Number of ligands	Number of models
**Tool: AF3**							
A2G	√	√	√	√	×	3	180
AC1	√	√	×	×	×	2	100
BGC	√	√	×	×	×	1	60
FUC	√	√	√	√	×	12	760
GAL	√	√	√	×	√	13	800
GLC	√	√	×	×	×	3	140
NDG	√	√	×	×	×	3	180
NAG	√	√	√	√	×	8	520
SIA	√	×	×	×	√	3	180
**Tool: Boltz_1x**							
A2G	×	×	×	×	√	3	180
FUC	×	×	×	×	√	12	760
GAL	×	×	×	×	√	13	800
NAG	×	×	×	×	√	8	520
SIA	√	×	×	×	√	3	180

a The total number of models is calculated by multiplying the number of input types, the number of cofolding types (with different proteins or glycan alone; Figure 3), and the number of random seeds.

In contrast to AF3, glycan modeling with Boltz-1x showed markedly improved stereochemical accuracy, consistent with previous reports highlighting its ability to correct ligand chirality.^15,17^ With Boltz-1x, 77.4% of predicted models were stereochemically correct, and chirality violations decreased to just 1%. The frequency of additional error types also declined substantially, and models rarely exhibited multiple co-occurring violations (Figure 1A and Figure S1). However, we detected a notable increase in improper structural configurations, particularly in *N*-acetyl-α-neuraminic acid (SIA) (Figure S1). Previous work by Huang et al.^3^ reported challenges in refining glycosidic linkages between SIA and galactose (GAL). But our findings extend this observation by revealing more pronounced stereochemical anomalies within the SIA unit itself, indicating that Boltz-1x may introduce a distinct class of structural errors in certain monosaccharides.

### Co-folding Method and Different Notation of the Carbohydrate Ligand Input Can Affect Ligand Implausibility of AF3 and Boltz-1x

Overall Boltz-1x produced fewer errors than AF3, but the stereochemical outcomes of each program were strongly influenced by the cofolding context in which glycans were modeled (Figure 2A). The number of errors produced by AF3 decreased when glycans were modeled in complex with their corresponding native protein partners, suggesting that the presence of a protein scaffold may provide additional structural constraints that reduce implausible ligand conformations. In contrast, Boltz-1x exhibited the highest accuracy when modeling free glycans. Notably, the presence of a control, nonglycan binding protein slightly reduced AF3’s error rate, relative to glycan-only predictions, but caused Boltz-1x to generate more errors than in the glycan-only condition (Figure 2A). These contrasting trends indicate that AF3 and Boltz-1x respond differently to cofolding environments, and that the influence of protein context on ligand stereochemistry differs, depending on the modeling framework.

**Figure 2:**
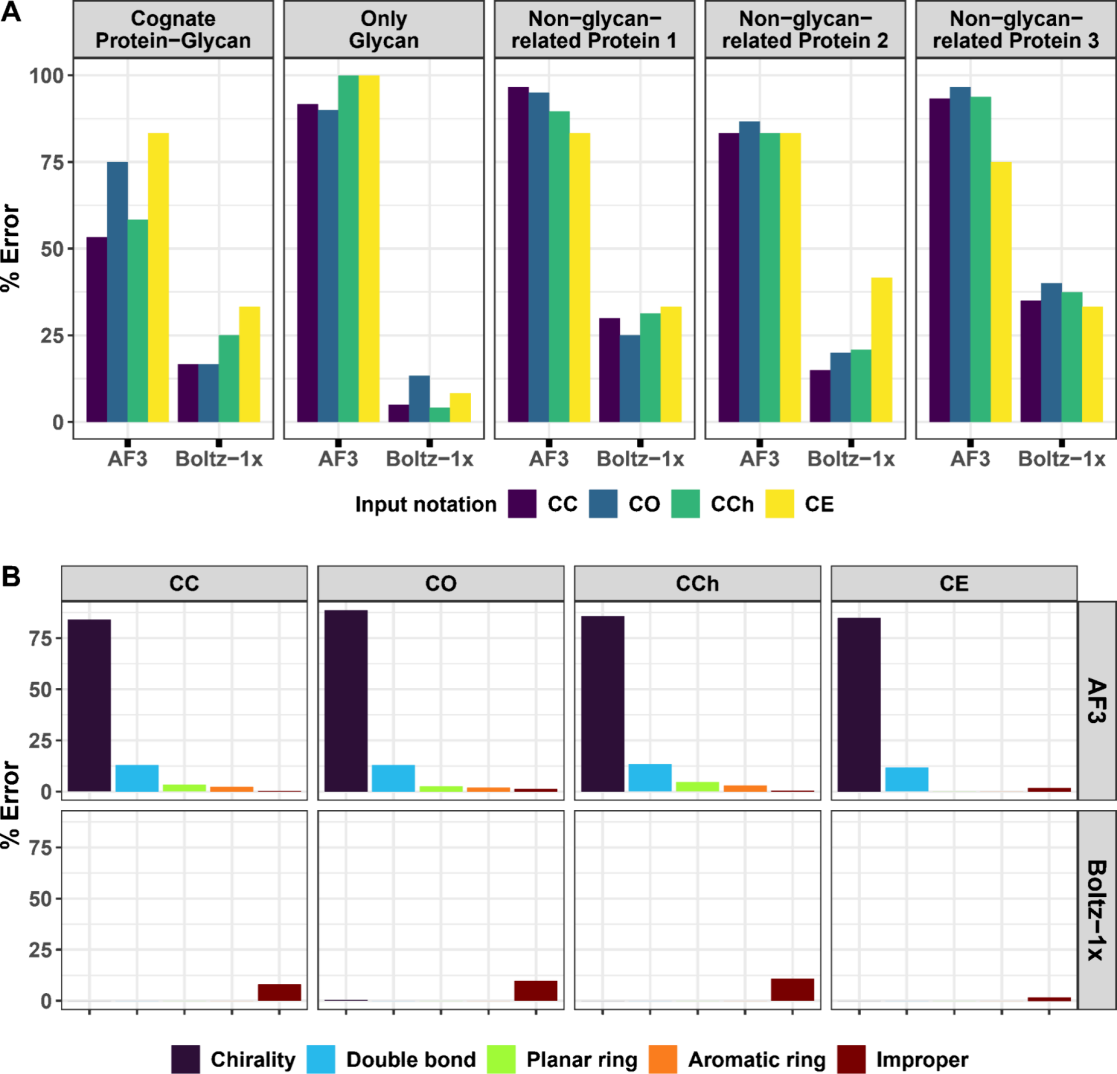
(A) Glycan stereochemistry errors across different modeling tools, types of Canonical SMILES inputs, and cofolding strategies. Each bar is color-coded according to the input notation, with CC, CO, CCh, and CE representing Canonical SMILES generated using CACTVS, OpenEye, ChEBI, and ChEMBL, respectively. The titles above each column group indicate the cofolding method applied, where Only glycan refers to modeling performed on free glycans without associated proteins, Protein-glycan refers to predictions involving native glycan–protein complexes, and the nonglycan-related protein complexes include glycans modeled with 1TGM (Control 1), 4CUT (Control 2), and 6DYR (Control 3). (B) The influence of different types of Canonical SMILES inputs and modeling tools on the percentage and diversity of structural errors in predicted glycan models. Each bar is color-coded according to the manual error categories. The titles above each column group indicate the input notation used, while the titles beside each row group specify the modeling tool used in the evaluation.

We next examined whether the choice of ligand SMILES input notation influenced stereochemical outcomes. Overall, input notation had only a minor effect on the number and type of errors produced (Figure 2B). Among the notations tested, Canonical SMILES generated using CACTVS (CC) appeared to be the most effective in reducing error rates, but this tentative conclusion requires further testing. Notably, the influence of input notation depended on both the modeling tool and the cofolding context (free glycans or glycan–protein complexes), indicating that these variables interact to shape stereochemical quality (Figure 2A and 2B). Across all input notations, AF3 consistently produced the highest frequency of chirality violations, followed by errors involving artificial double bonds, planar rings, aromatic-like rings, and improper monosaccharide configurations. In contrast, Boltz-1x largely eliminated chirality, double-bond, and ring-flattening errors but generated a higher number of improper structural configurations compared to AF3. Figure 3 provides a detailed overview of the errors identified for each ligand, categorized by cofolding tool, cofolding method, and SMILES input notation.

### Influence of Monosaccharide Identity on Error Profiles

We next examined whether the identity of individual monosaccharide units influenced the types and frequencies of stereochemical errors. Because some sugars are less common in glycoscience datasets and therefore sparsely represented in structural databases, we hypothesized that such monosaccharides may be more susceptible to modeling errors than well-characterized counterparts. For instance, the common beta-d-glucopyranose (BGC) might be expected to yield fewer structural violations than the more chemically modified 2-acetamido-2-deoxy-beta-d-glucopyranose (NAG).

This trend was reflected in AF3 predictions. Specifically, NAG-containing glycans exhibited a broader range of error types than those containing BGC. However, this observation must be interpreted cautiously. In our dataset, 8 out of the 15 glycans contained NAG, whereas only one glycan included BGC. Thus, the greater diversity of errors associated with NAG may partly reflect its higher representation rather than an inherently greater vulnerability to modeling errors (Table 1).

When comparing sugars with equal representation, such as NDG and GLC, each found in 3 out of 15 glycans, the number and types of errors were comparable. Similarly, beta-d-galactopyranose (GAL) and alpha-l-fucopyranose (FUC) were each associated with four out of five distinct error types, although the specific error categories differed. Interestingly, despite being a more commonly studied sugar, GAL exhibited a greater diversity of error categories than *N*-acetyl-alpha-neuraminic acid (SIA). Additionally, we observed a notable tendency for AF3 to introduce double bond errors. Nearly all monosaccharide models generated by AF3 exhibited at least one occurrence of this error type (Table 1), although the frequency was lower than chirality issues (Figure 3).

**Figure 3:**
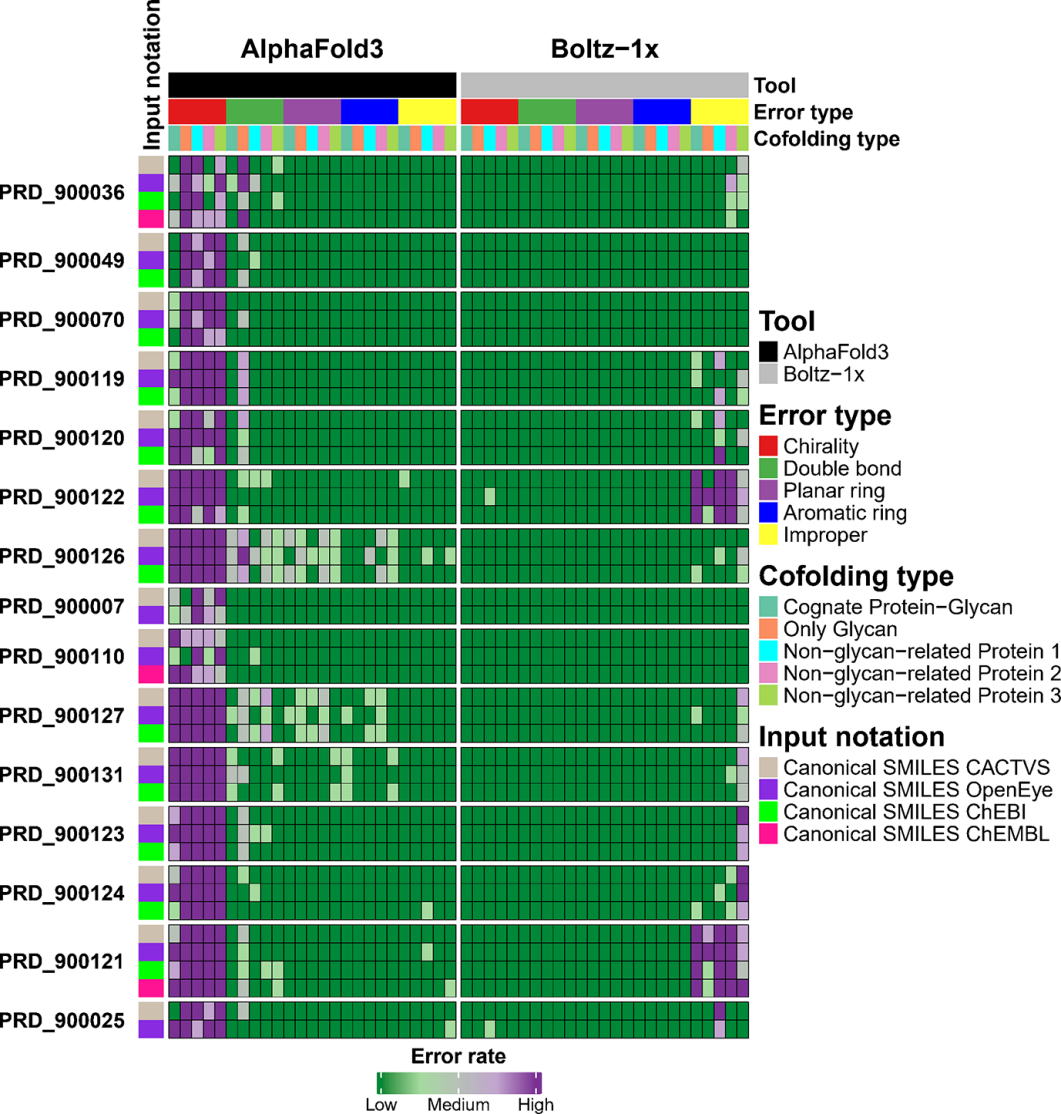
A heatmap providing an overview of the stereochemistry errors identified in Figure 2. The error rates are organized according to the ligand used, the modeling tool applied, the input notation provided, the cofolding method employed, and the specific types of errors detected.

For Boltz-1x, the hypothesis also appears valid, as less frequently encountered or structurally complex monosaccharides generally exhibited a higher incidence of stereochemical errors, although exceptions such as BGC and GLC were noted. As shown in Table 1 and Figures 2B and 3, Boltz-1x substantially reduced chirality-related and ring-flattening errors compared to AF3,^15^ yet it introduces a distinct category of inaccuracies involving improper monosaccharide configurations (Figure S1). This newly identified error type, particularly evident in sugars such as N-acetyl-α-neuraminic acid (SIA) has not been previously reported, suggesting that care should be taken when using Boltz-1x to generate models.

### Use of Alternative Approaches and Analysis of Published Datasets Reveals Further Problems

Among new strategies aimed at overcoming AF3’s stereochemical limitations, a notable recent advance involves the use of BondedAtomPairs (BAP) syntax, which explicitly encodes glycan connectivity and has been reported to produce highly consistent and stereochemically accurate glycan structures in AF3.^3^ Because BAP syntax requires detailed glycobiological knowledge to define correct monosaccharide types and linkages, the developers introduced JAAG (JSON input file Assembler for AlphaFold 3 with Glycan integration), a web-based graphical interface that automatically generates AF3 JSON files containing BAP syntax.^18^ This approach greatly simplifies BAP usage and has been widely promoted as a solution to AF3’s glycan stereochemistry issues.

We modeled 13 of our 15 glycans in the absence of protein (technical limitations prevented modeling of PRD_900007 and PRD_900110) and found that stereochemical errors were absent. However, we identified a previously unreported limitation of this approach, similarly to Boltz-1x, which corrected many stereochemical issues but introduced new structural anomalies. BAP syntax consistently produced a systematic structural error: the terminal atom of the first monosaccharide (the reducing end) was removed in every model (Figure 4A). We also examined the occurrence of errors in the model datasets accompanying the BAP publication,^3^ and confirmed that they also showed the same reducing-end deletion (https://modelarchive.org/doi/10.5452/ma-af3glycan). Surprisingly, these models also contained a variety of additional chirality and improper errors (Figure 4B, Tables S3 and S4).

**Figure 4:**
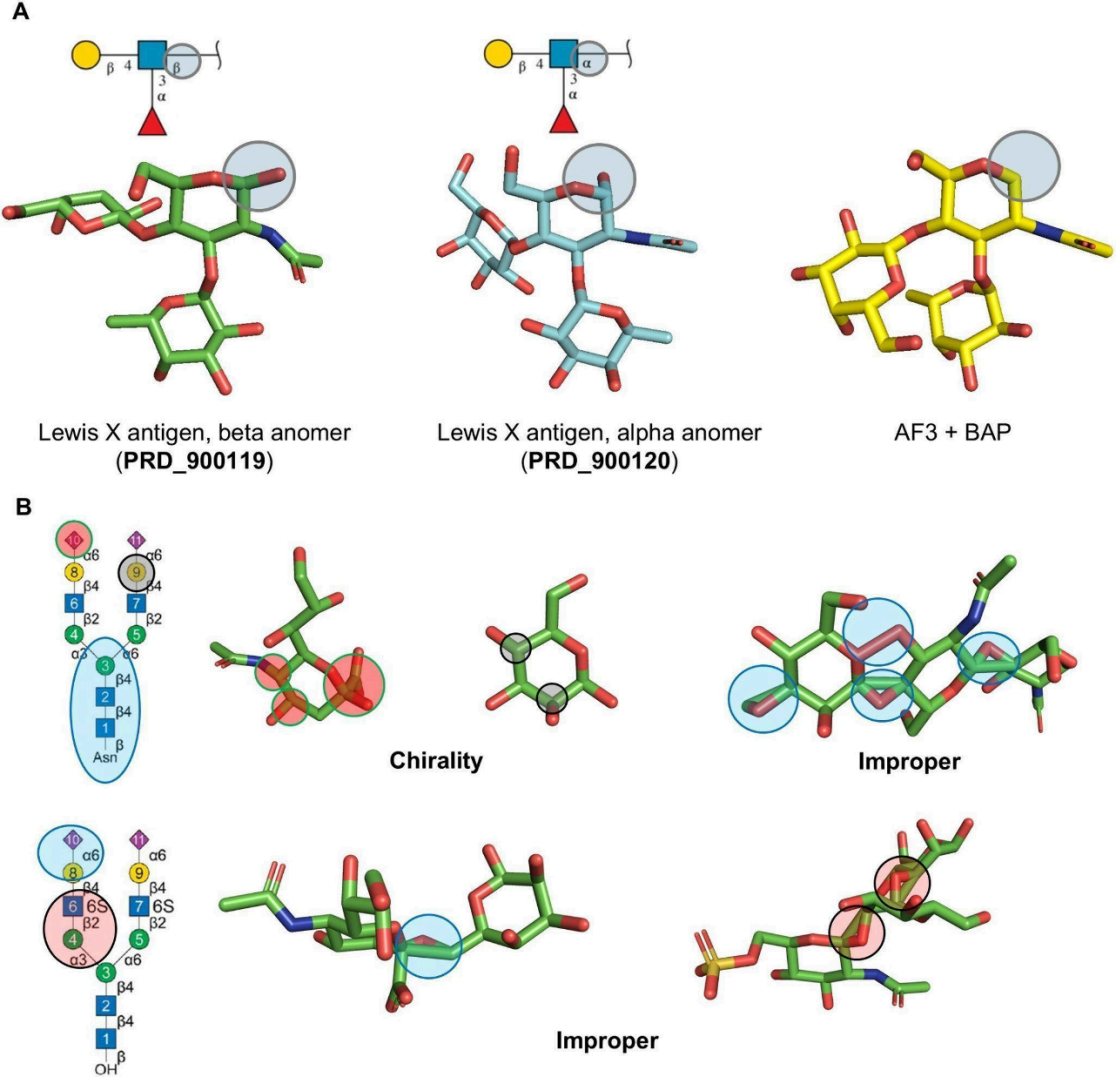
While BAP syntax successfully resolved all stereochemical issues encountered with our set of 15 ligands, it introduced a new anomaly by removing the terminal atom of the first monosaccharide unit in the oligosaccharide chain (the reducing end). This issue is evident when AF3 using BAP syntax produces the same results for antigen pairs that differ only in their α- and β- anomer (A). Manual inspection of the original AF3 + BAP databset revealed that stereochemical errors, including chirality issues and improper bond configurations, are still evident (see also Tables S3 and S4) (B).

Finally, we analyzed AF3 models of complexes for the Benchmark of CArbohydrate Protein Interactions (BCAPIN) set of experimental structures.^16^ As shown in Table S5, although the models were high quality (scoring ≥0.80) by the authors’ newly developed DockQC measure, chirality issues were found among the models for most glycans. Together with the BAP results in Tables S3 and S4, these new datasets add monosaccharides XYP MAN BMA GLA as well as sulfated sugars to those analyzed in our set of 15 glycans and demonstrate the occurrence of stereochemical problems in MAN, BMA and sulfated sugars. They also illustrate the occurrence of errors in covalently linked glycans.^3^

## Discussion

AF3 originally reported a chirality violation rate of only 4.4% across its full prediction set.^1^ However, subsequent studies revealed much higher rates under specific conditions, 30%–40% for protein–ligand complexes 4 and 51% for d-peptides.^14^ Our findings of 900 AF3-predicted oligosaccharide structures reveal an even more severe limitation: 85.78% of glycan models display stereochemical errors. These errors were not only limited to incorrect stereocenter assignments but also the introduction of artificial double bonds, planar and aromatic ring geometries, and improper monosaccharide configurations. These violations frequently co-occurred within individual sugar units and, in some cases, propagated across multiple residues (Figure 1B).

Several factors contribute to the pronounced failure of AF3 in glycan modeling. First, carbohydrate-containing structures constitute only ∼5.7% of the PDB (∼14 000 PDB entries),^12^ suggesting that AF3’s training data provide limited glycan representation, particularly for multiresidue oligosaccharides. Second, AF3 may exhibit learned biases toward planar ring geometries derived from nucleobase-rich training data,^4^ which could promote flattening of pyranose rings. Third, AF3’s stereochemistry validation relies on PoseBusters, which was benchmarked primarily on protein–ligand datasets (308 internal and 85 external complexes) rather than glycans.^19^ Consequently, PoseBusters may misclassify glycan stereochemistry. As emphasized by Huang et al.,^3^ no robust automated tool exists for glycan-specific structural assessment, limiting high-throughput evaluation. In this context, manual inspection remains the most reliable but highly labor-intensive approach for verifying glycan models.

Boltz-1x^15^ substantially improved stereochemical plausibility relative to AF3, reducing total error frequency to 22.6% and nearly eliminating chirality violations. However, our results reveal a previously unreported limitation: Boltz-1x introduced a distinct class of improper monosaccharide conformations, particularly in SIA (Figure 2B and Figure S1). Although these errors are fewer in number than AF3’s, their presence indicates that corrections targeting one failure mode may inadvertently create new structural artifacts.

The most recent approach, BAP syntax,^3^ effectively eliminated all AF3 stereochemical errors in our dataset. Yet, manual inspection uncovered a systematic and previously unrecognized anomaly: BAP consistently removes the reducing-end atom from the first monosaccharide in every model. This seemingly small modification can have major implications for glycan identity and annotation,^20^ and can impact computational analyses, such as glycan bioinformatics workflows,^21^ Lewis antigen modeling,^22,23^ glycan-antibody specificity prediction,^24^ and lectin–glycan binding prediction.^25^ Importantly, inspection of the publicly available BAP-generated models from the original study confirmed that the reducing-end deletion is systematic and not dataset-specific. The same set of models also contain chirality and improper errors not seen in our BAP results (see Figure 4B, Tables S3 and S4).

Together, these findings reveal that all current AF3-compatible glycan modeling approaches, AF3 alone, Boltz-1x, and BAP syntax, exhibit significant limitations, including two previously undocumented structural anomalies. While some of these could potentially be resolved during onward use in molecular dynamics simulations, for example, others such as missing atoms and chirality errors would not. Given the absence of automated, glycan-aware stereochemical validation tools, manual inspection remains essential for accurate evaluation but is impractical for high-throughput modeling. Future development should therefore focus not only on improving modeling accuracy but also on creating dedicated, automated frameworks for glycan quality assessment. Such tools will be critical for ensuring that next-generation AI-based structure predictors can reliably support glycan-focused biology, immunology, and biotechnology.

## Supplementary Material



## Data Availability

All models generated by AF3 and Boltz-1x used in this study are available at: https://github.com/mluthfichula/glycan_modeling_github.git

## Notes

Section S1 (Methodology): covers datasets used, modeling methods and scoring; Figure S1: Illustrative examples of Boltz-1x structural violations; Table S1: Main set of glycans studies here, BIRD IDs, PDB origins, glycan names and 2D diagrams; Table S2: Privateer reports for sample errors detected by manual observation; Table S3: Errors found in outputs from Huang et al.;^3^ Table S4: Errors found in further outputs from Huang et al.;^3^ Table S5: Errors found in outputs from Canner et al.^16^ (PDF)
