# Supplementary material for: Physical Implausibility of Carbohydrate Ligands in Results of Deep Learning-Based Cofolding Methods
Source: J Chem Inf Model. 2026 Mar 23;66(7):3456–63. doi: 10.1021/acs.jcim.5c03075 (PMC13080964; doi:10.1021/acs.jcim.5c03075)
Supplement: Supplementary file 1 [file ci5c03075_si_001.pdf]

Supporting Information
Physical implausibility of carbohydrate ligands in results of deep learning-based cofolding methods

Muhammad Luthfi[1,2], Adam J Simpkin[2], Luc G Elliott[2], Pornthep Sompornpisut[3]*, and Daniel J. Rigden[2]*

[1]*Program in Bioinformatics and Computational Biology, College of Interdisciplinary and Integrative Studies, Chulalongkorn University, Bangkok, 10330, Thailand*

[2]*Department of Biochemistry, Cell and Systems Biology; Institute of Structural, Molecular and Integrative Biology, University of Liverpool, Crown Street, Liverpool L69 7ZB, United Kingdom*

[3]*Center of Excellence in Computational Chemistry, Department of Chemistry, Chulalongkorn University, Bangkok, 10330, Thailand*

*To whom correspondence can be addressed: drigden@liverpool.ac.uk

S1 Methodology

S1.1 Dataset

This study began by examining a set of oligosaccharides previously studied with fucose-specific lectins from *Ralstonia solanacearum* and *Burkholderia ambifaria*, which have been commonly used in glycan-binding protein and molecular docking studies.[1,2] Since only five unique oligosaccharides were identified from these sources, the dataset was expanded to incorporate additional lectin-binding and non-binding glycan from diverse biological origins in order to increase structural and stereochemical diversity. This resulted in a final set of 15 ligands (Table S1), all of which are experimentally determined in crystal structures in the Protein Data Bank (PDB).[3] To further broaden the diversity of sugar units, three additional datasets were incorporated: Siglec-2 (CD22) structures multiply modified with 11 N-linked G2S2 glycans (with or without addition of a further non-covalently-linked glycan),[4] and models of a high-quality dataset of experimental structures – Benchmark of CArbohydrate Protein Interactions (BCAPIN) - from which ligands with high DockQC scores (≥ 0.80) were selected.[5]

S1.2 Prediction Methodology

Structural modeling was carried out using AlphaFold 3[6] and Boltz-1x[7], both implemented through the ABCFold platform[8]. Input data for ABCFold were organized into three categories: (i) protein sequences paired with their known binding oligosaccharides, (ii) oligosaccharides modelled in the absence of protein, and (iii), as a control, non-carbohydrate-binding proteins (PDB entries 1TGM, 4CUT, and 6DYR) combined with each oligosaccharide. All protein sequences were modelled as monomers. Because both AlphaFold 3 and Boltz-1x accept SMILES strings for ligand input without specifying a preferred canonical form, we evaluated whether different Canonical SMILES representations (CACTVS, OpenEye, ChEBI, and ChEMBL) obtained from PDB and related chemical databases influenced the predicted oligosaccharide structures. To assess reproducibility and quantify variability, four independent modeling runs with different seeds were conducted.

S1.3 Metrics

As there are currently no appropriate, validated, and automated tools for carbohydrate ligand quality assessment,[4] stereochemistry evaluation in this study was performed manually. If a single atom within an oligosaccharide violated stereochemical rules, the entire oligosaccharide was classified as failing the stereochemistry evaluation. The stereochemistry error rate for an oligosaccharide was calculated by dividing the number of oligosaccharides that failed the evaluation by the total number of replicates (or models generated), then multiplying by 100%. To support and validate the manual inspection, particularly for detecting aromatic ring and planar ring errors, a subset of models failing these criteria was additionally evaluated by manual renaming of atoms in the original output and use of Privateer.[9]

S1.4 Software for Data Analysis and Visualization

Data processing, stereochemical inspection, and visualization were performed using Microsoft Excel, PyMOL v2.5.0 Open-Source, and R v4.5.2. R packages included tidyverse, viridis, patchwork, corrplot, matrixStats, janitor, ComplexHeatmap, gridExtra, cowplot, grid, circlize, and RColorBrewer.

S2 Supplementary figures

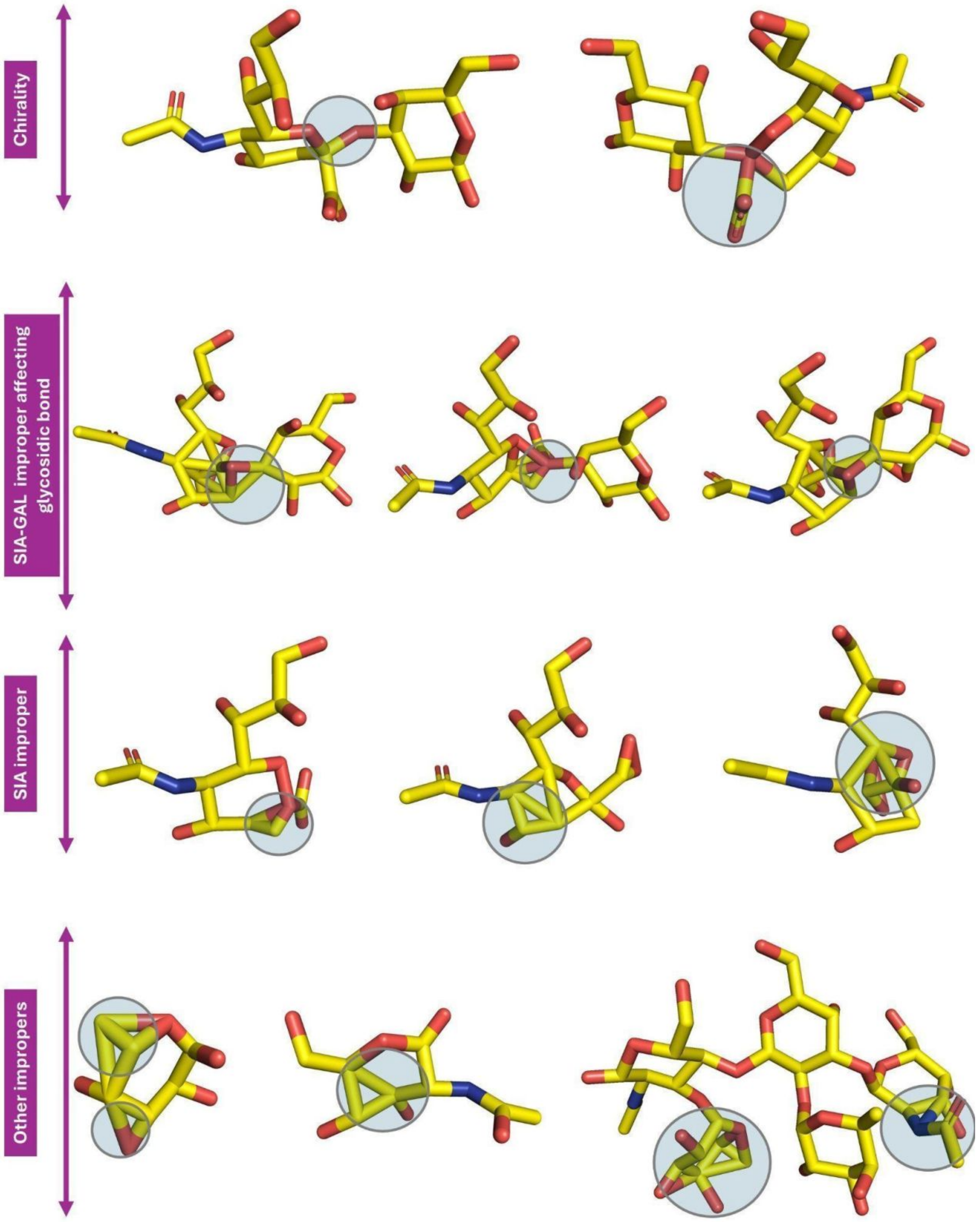

Figure S1. Structural violations observed in glycan models generated by Boltz-1x. N-acetyl-α-neuraminic acid (SIA) was the primary contributor to the 22.56% of models classified as implausible. In addition to challenges in modeling glycosidic linkages between SIA and galactose (GAL) as previously reported by Huang et al.,[4] we identified multiple structural anomalies within SIA itself, extending beyond linkage issues and manifesting as various forms of improper stereochemical violations.

S3 Supplementary tables

Table S1. Glycan based on Biologically Interesting Molecule Reference Dictionary (BIRD) ID and PDB identifiers used as input for modeling protein-glycan complexes with AlphaFold 3 and Boltz-1x

BIRD ID	PDB ID	Glycan Name	2D Diagram
PRD_900036	3ZZV	H type 2 antigen, beta anomer	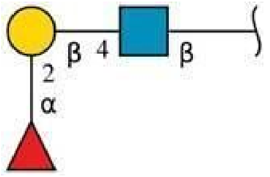
PRD_900049	3ZW2	type 1 antigen, beta anomer	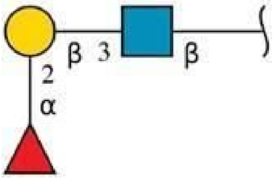
PRD_900070	2BS5	2'-fucosyllactose	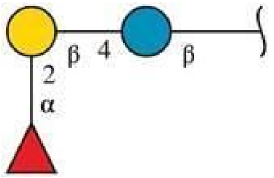
PRD_900119	5AJB	Lewis X antigen, beta anomer	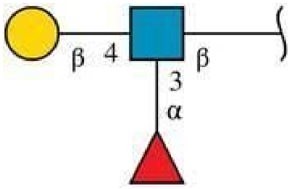
PRD_900120	5AJC	Lewis X antigen, alpha anomer	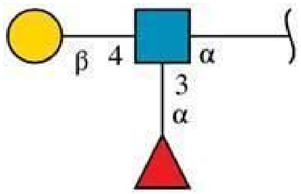
PRD_900122	5AJC	Sialyl-Lewis X antigen, alpha anomer	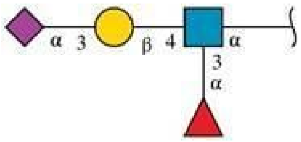

PRD_900126	5F7N	Blood group A Lewis B antigen, beta anomer	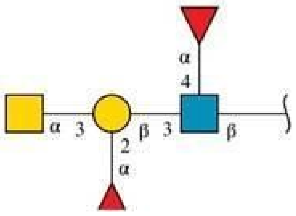
PRD_900007	6M6T	Alpha-acarbose	Not applicable
PRD_900110	6M6T	Acarbose-derived trisaccharide	Not applicable
PRD_900127	2WMK	Blood group A Lewis Y antigen, beta anomer	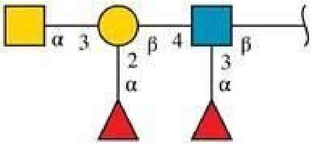
PRD_900131	3ASP	Blood group A H type 1 antigen, beta anomer	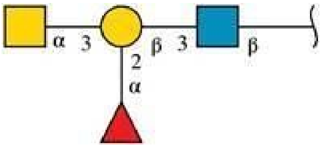
PRD_900123	4GWJ	Lewis B antigen, beta anomer	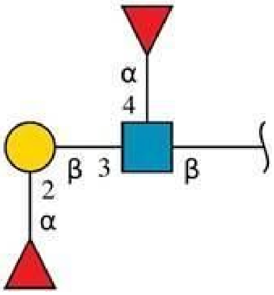
PRD_900124	4D4U	Lewis Y antigen, alpha anomer	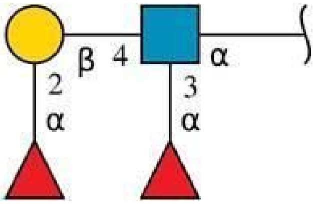
PRD_900121	4USO	Sialyl-Lewis X antigen, beta anomer	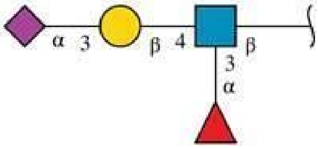

PRD_900025	1QFO	3'-sialyl-alpha-lactose	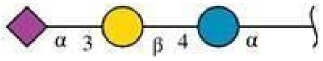

Table S2. Validation of manual inspection results for planar and aromatic ring errors by observing violations using Privateer.

Error type	Sugar unit	Privateer report	Checked structure
Planar ring	A2G	Number of issues:<br>a. conformation = 1<br>b. anomer = 1<br>c. pucker = 1	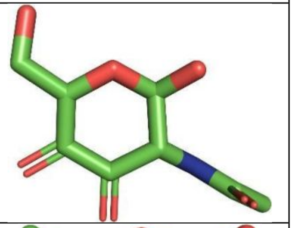
	FUC	Number of issues:<br>a. conformation = 1<br>b. anomer = 1<br>c. pucker = 1<br>d. chirality = 1	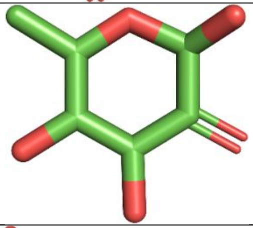
	NAG	Number of issues:<br>a. conformation = 1<br>b. anomer = 1<br>c. pucker = 1	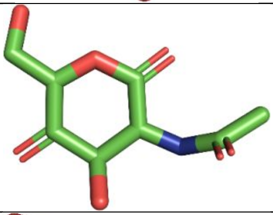
	GAL	Number of issues:<br>a. conformation = 1<br>b. pucker = 1	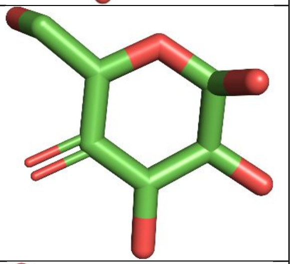
Aromatic ring	A2G	Number of issues:<br>a. conformation = 1<br>b. pucker = 1<br>c. chirality = 1	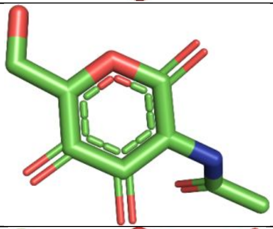
	FUC	Number of issues:<br>a. conformation = 1<br>b. anomer = 1<br>c. pucker = 1<br>d. chirality = 1	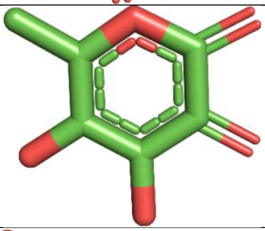
	NAG	Number of issues:<br>a. conformation = 1<br>b. anomer = 1<br>c. pucker = 1	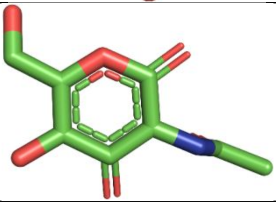

Table S3. Manual inspection of stereochemistry errors in two structures of Siglec-2 (CD22), generated using the AF3 + BAP syntax method, each covalently modified at 11 different sites with G2S2 N-linked glycans

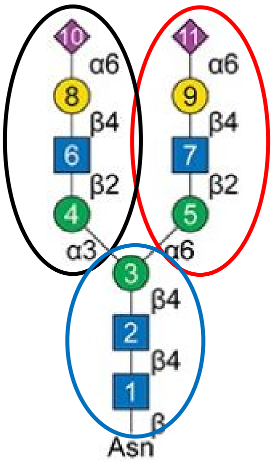

Note:

Region 1

Region 2

Region 3

Ligand number	Region 1	Region 2		Region 3	
	Improper	Improper	Chirality	Improper	Chirality
1	□	□	□	BMA-MAN	□
2	□	MAN-NAG, NAG-GAL, GAL-SIA	GAL	GAL-SIA	GAL
3	□	GAL-SIA	□	BMA-MAN, MAN-NAG, NAG-GAL	GAL
4	□	BMA-MAN	GAL	□	GAL
5	□	GAL-SIA	□	BMA-MAN, MAN-NAG	GAL
6	□	GAL-SIA	□	BMA-MAN	GAL
7	□	□	□	BMA-MAN	GAL
8	□	BMA-MAN, MAN-NAG,	□	BMA-MAN	GAL

		GAL-SIA			
9	□	□	GAL	BMA-MAN	GAL
10	□	GAL-SIA	□	BMA-MAN, MAN-NAG, NAG-GAL	GAL
11	□	BMA-MAN	□	□	GAL
12	NAG-NAG, NAG-BMA	BMA-MAN	□	□	GAL
13	NAG-NAG	BMA-MAN, MAN-NAG, GAL-SIA	□	BMA-MAN	GAL
14	□	□	□	BMA-MAN	□
15	□	□	□	□	□
16	□	□	□	□	□
17	□	GAL-SIA	□	BMA-MAN	□
18	□	GAL-SIA	□	□	GAL
19	□	□	□	□	GAL
20	□	□	□	□	□
21	□	GAL-SIA	□	BMA-MAN	□
22	□	MAN-NAG	SIA	□	GAL

Table S4. Manual inspection of stereochemistry errors in two structures of Siglec-2 (CD22), generated using the AF3 + BAP syntax method, each covalently modified at 11 different sites with G2S2 N-linked glycans and including an additional 6-O-sulfated

GlcNac (GlcNAc6S).

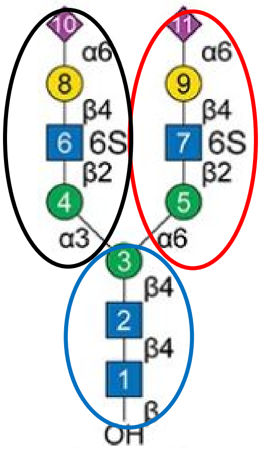

Note:

Region 1

Region 2

Region 3

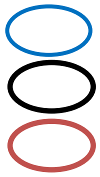

Ligand number	Region 1	Region 2		Region 3	
	Improper	Improper	Chirality	Improper	Chirality
1 (GlcNAc6S)	□	BMA-MAN, MAN-NAG, GAL-SIA	GAL	□	□
2	NAG-NAG, NAG-BMA	BMA-MAN, MAN-NAG, GAL-SIA	GAL	BMA-MAN, MAN-NAG	□
3	□	BMA-MAN, GAL-SIA	□	BMA-MAN	□
4	□	GAL-SIA	GAL	BMA-MAN	□
5	□	BMA-MAN, MAN-NAG, GAL-SIA	□	BMA-MAN	□
6	□	BMA-MAN, MAN-NAG, GAL-SIA	□	BMA-MAN	□
7	□	BMA-MAN, GAL-SIA	□	□	□

8	□	□	□	□	□
9	□	BMA-MAN, MAN-NAG, GAL-SIA	GAL	□	□
10	□	BMA-MAN, MAN-NAG, GAL-SIA	□	BMA-MAN	□
11	□	GAL-SIA	GAL	BMA-MAN	□
12	NAG-NAG, NAG-BMA	BMA-MAN, MAN-NAG, NAG-GAL, GAL-SIA	□	BMA-MAN	□
13	NAG-BMA	BMA-MAN, MAN-NAG, GAL-SIA	□	BMA-MAN	□
14	NAG-NAG,	MAN-NAG, GAL-SIA	□	BMA-MAN	□
15	□	□	GAL	□	□
16	□	□	□	□	□
17	□	BMA-MAN, MAN-NAG, GAL-SIA	□	□	□
18	□	BMA-MAN, MAN-NAG, GAL-SIA	GAL	BMA-MAN	□
19	□	BMA-MAN, MAN-NAG, GAL-SIA	GAL	BMA-MAN	□

20	□	□	□		□
21	□	BMA-MAN, MAN-NAG, GAL-SIA	□		□
22	□	BMA-MAN, MAN-NAG, GAL-SIA	GAL	BMA-MAN	□
23	□	BMA-MAN, MAN-NAG, GAL-SIA	GAL	BMA-MAN	□

Table S5. Manual inspection of stereochemistry errors in 38 AF3 models from the BCAPIN dataset that have high DockQC metric scores (≥ 0.80).

PDB ID	Rank	DockQC score	Ligand class	Unit sugar (s)	Stereochemistry issue	Note
7BLG	0	0.941	Monosaccharide	GAL	Yes	Chirality
	1	0.964				
	2	0.949			No	NA
	3	0.948				
	4	0.974				
7EXO	0	0.946	Monosaccharide	BMA	No	NA
	1	0.939				
	2	0.948				
	3	0.95				
	4	0.945				
7JNF	0	0.955	Monosaccharide	A2G	No	NA
	1	0.984			Yes	Chirality
	2	0.954			No	NA
	3	0.992				

	4	0.991				
7JWF	0	0.961	Disaccharide	GAL, GLA	Yes	Chirality (GAL)
	1	0.956				
	2	0.963				
	3	0.955				
	4	0.957				
7VI7	0	0.965	Monosaccharide	NDG	Yes	Chirality
	1	0.96			No	NA
	2	0.962			Yes	Chirality
	3	0.963			No	NA
	4	0.964			Yes	Chirality
7W11	0	0.882	Monosaccharide	GLC	Yes	Chirality
	1	0.91				
	2	0.809				
	3	0.875			No	NA
	4	0.871				
8BF3	2	0.893	Disaccharide	XYP	No	NA
	3	0.819				

	4	0.939				
8D0R	0	0.968	Oligosaccharide	NAG, GAL, FUC	No	NA
	1	0.961				
	2	0.931				
	3	0.972				
	4	0.977				

REFERENCES

(1) Mishra, S. K.; Adam, J.; Wimmerová, M.; Koča, J. In Silico Mutagenesis and Docking Study of Ralstonia Solanacearum RSL Lectin: Performance of Docking Software To Predict Saccharide Binding. *J. Chem. Inf. Model.* **2012**, *52* (5), 1250–1261. https://doi.org/10.1021/ci200529n.

(2) Topin, J.; Arnaud, J.; Sarkar, A.; Audfray, A.; Gillon, E.; Perez, S.; Jamet, H.; Varrot, A.; Imberty, A.; Thomas, A. Deciphering the Glycan Preference of Bacterial Lectins by Glycan Array and Molecular Docking with Validation by Microcalorimetry and Crystallography. *PloS One* **2013**, *8* (8), e71149. https://doi.org/10.1371/journal.pone.0071149.

(3) Vallat, B.; Rose, Y.; Piehl, D. W.; Duarte, J. M.; Bittrich, S.; Bi, C.; Segura, J.; Zalevsky, A.; Sekharan, M. R.; Webb, B. M.; Bhikadiya, C.; Voigt, M.; Henry, J.; Chao, H.; Pingale, A.; Raymond, D.; Sagendorf, J.; Peisach, E.; Feng, Z.; Guranovic, V.; Smith, J.; Rose, A. S.; Young, J. Y.; Berman, H. M.; Sali, A.; Burley, S. K. RCSB Protein Data Bank: Delivering Integrative Structures alongside Experimental Structures and Computed Structure Models. *Nucleic Acids Res.* **2025**, gkaf1187. https://doi.org/10.1093/nar/gkaf1187.

(4) Huang, C.; Kannan, N.; Moremen, K. W. Modeling Glycans with AlphaFold 3: Capabilities, Caveats, and Limitations. *Glycobiology* **2025**, *35* (10), cwaf048. https://doi.org/10.1093/glycob/cwaf048.

(5) Canner, S. W.; Lu, L.; Takeshita, S. S.; Gray, J. J. Evaluation of De Novo Deep Learning Models on the Protein-Sugar Interactome. bioRxiv September 6, 2025, p 2025.09.02.673778. https://doi.org/10.1101/2025.09.02.673778.

(6) Abramson, J.; Adler, J.; Dunger, J.; Evans, R.; Green, T.; Pritzel, A.; Ronneberger, O.; Willmore, L.; Ballard, A. J.; Bambrick, J.; Bodenstein, S. W.; Evans, D. A.; Hung, C.-C.; O'Neill, M.; Reiman, D.; Tunyasuvunakool, K.; Wu, Z.; Žemgulytė, A.; Arvaniti, E.; Beattie, C.; Bertolli, O.; Bridgland, A.; Cherepanov, A.; Congreve, M.; Cowen-Rivers, A. I.; Cowie, A.; Figurnov, M.; Fuchs, F. B.; Gladman, H.; Jain, R.; Khan, Y. A.; Low, C. M. R.; Perlin, K.; Potapenko, A.; Savy, P.; Singh, S.; Stecula, A.; Thillaisundaram, A.; Tong, C.; Yakneen, S.; Zhong, E. D.; Zielinski, M.; Žídek, A.; Bapst, V.; Kohli, P.; Jaderberg, M.; Hassabis, D.; Jumper, J. M. Accurate Structure Prediction of Biomolecular Interactions with AlphaFold 3. *Nature* **2024**, *630* (8016), 493–500. https://doi.org/10.1038/s41586-024-07487-w.

(7) Wohlwend, J.; Corso, G.; Passaro, S.; Getz, N.; Reveiz, M.; Leidal, K.; Swiderski, W.; Atkinson, L.; Portnoi, T.; Chinn, I.; Silterra, J.; Jaakkola, T.; Barzilay, R. Boltz-1 Democratizing Biomolecular Interaction Modeling. bioRxiv May 6, 2025, p 2024.11.19.624167. https://doi.org/10.1101/2024.11.19.624167.

(8) Elliott, L. G.; Simpkin, A. J.; Rigden, D. J. ABCFold: Easier Running and Comparison of AlphaFold 3, Boltz-1, and Chai-1. *Bioinforma. Adv.* **2025**, *5* (1), vbaf153. https://doi.org/10.1093/bioadv/vbaf153.

(9) Dialpuri, J. S.; Bagdonas, H.; Atanasova, M.; Schofield, L. C.; Hekkelman, M. L.; Joosten, R. P.; Agirre, J. Analysis and Validation of Overall N-Glycan Conformation in Privateer. *Acta Crystallogr. Sect. Struct. Biol.* **2023**, *79* (6), 462–472. https://doi.org/10.1107/S2059798323003510.
